# Supplementary material for: The Performances of SNAQ, GLIM, mNICE, and ASPEN for Identification of Neurocritically Ill Patients at High Risk of Developing Refeeding Syndrome
Source: Nutrients. 2022 Sep 28;14(19):4032. doi: 10.3390/nu14194032 (PMC9572145; doi:10.3390/nu14194032)
Supplement: Supplementary file 1 [file nutrients-14-04032-s001.zip › nutrients-1891861-supplementary.pdf]

Supplementary Materials

Table S1. Short Nutritional Assessment Questionnaire (SNAQ).

Constant	Score
Did you lose weight unintentionally?	
More than 6 kg in the last 6 months	3
More than 3 kg in the last month	2
Did you experience a decreased appetite over the last month?	1
Did you use supplemental drinks or tube feeding over the last month?	1

Total of 0-1 point: no risk of malnutrition. 2 points: average risk of malnutrition. 3-5 points: high risk of malnutrition. In this study, patients were classified as being at high risk of RFS when scored SNAQ ≥ 2 scores.

Table S2. Phenotypic and etiologic criteria for the diagnosis of malnutrition of GLIM.

Phenotypic criteria*			Etiologic criteria*	
Weight loss (%)	Low BMI (kg/m^2)	Reduced muscle mass	Reduced food intake or assimilation	Inflammation
> 5% within past 6 months, or >10% beyond 6 months	< 20 if < 70 years, or < 22 if > 70 years<br>Asia:<br>< 18.5 if < 70 years, or < 20 if > 70 years	Reduced by validated body composition measuring techniques	≤ 50% of energy requirements > 1 week, or any reduction for > 2 weeks, or any chronic gastrointestinal condition that adversely impacts food assimilation or absorption	Acute disease/injury, or chronic disease-related

*Requires at least 1 phenotypic criterion and 1 etiologic criterion for diagnosis of malnutrition. All grey overlays had not been utilized for scoring in the table.

Table S3. Thresholds for severity grading of malnutrition into stage 1 (moderate) and stage 2 (severe) malnutrition according to GLIM.

	Phenotypic criteria			
	Weight loss (%)	Low BMI (kg/m^2)	Reduced muscle mass	Score
**Stage 1/Moderate malnutrition** (requires 1 phenotypic criterion that meets this grade)	5–10% within the past 6 months, or 10–20% beyond 6 months	< 20 if < 70 years, < 22 if ≥ 70 years	Mild to moderate deficit	1
**Stage 2/Severe malnutrition** (requires 1 phenotypic criterion that meets this grade)	> 10% within the past 6 months, or > 20% beyond 6 months	< 18.5 if < 70 years, < 20 if ≥ 70 years	Severe deficit	2

Patients were classified as being at high risk of RFS when scored GLIM ≥ 2.
All grey overlays had not been utilized for scoring in the table.

Table S4. The mNICE Guidelines for management and prevention of RFS in medical inpatients receiving nutritional therapy.

1. Initial Risk Assessment		
Minor risk factors	Major risk factor	Specific patient population at high risk<br>(careful assessment is recommended)

BMI < 18.5 kg/m^2	BMI < 16 kg/m^2	
Unintentional weight loss > 10% in the last 3-6 months	Unintentional weight loss > 15% within the last 3-6 months	Hunger strike, chronic severe dieting
Little or no nutritional intake for > 5 days	Little or no nutritional intake for > 10 days	History of bariatric surgery, short bowel syndrome
History of alcohol abuse or drugs including insulin, chemotherapy, antacids, or diuretics	Low baseline levels of potassium, phosphate, or magnesium before feeding	Tumor patients, frail elderly patients with chronic debilitating

BMI, body mass index; Low baseline levels of potassium, phosphate, or magnesium before feeding were defined as the serum levels less than 3.5 mmol/L (3.5 mg/dL), 0.8 mmol/L (2.5 mg/dL), and 0.74 mmol/L (1.8 mg/dL), respectively, based on hospital reference for adults.

Table S5. Risk Stratification of mNICE.

No risk (1 score)	Low risk (2 scores)	High risk (3 scores)	Very high risk (4 scores)
	1 minor risk factor	1 major or 2minor risk factors	BMI < 14kg/m^2; Weight loss > 20%; Starvation > 15 day

Patients were classified as being at high risk of RFS when scored mNICE ≥ 3 scores.

Table S6. ASPEN Consensus Criteria for Identifying Adult Patients at Risk for Refeeding Syndrome.

	**Moderate risk: 2 risk criteria needed (1 score)**	**Significant risk: 1 risk criteria needed (2 scores)**
BMI	16-18.5 kg/m^2	< 16 kg/m^2
Weight loss	5% in 1 month	7.5% in 3 months or > 10% in 6 months
Caloric intake	None or negligible oral intake for 5-6 days OR < 75% of estimated energy requirement for > 7 days during an acute illness or injury OR < 75% of estimated energy requirement for > 1 month	None or negligible oral intake for > 7 days OR < 50% of estimated energy requirement for > 5 days during an acute illness or injury OR < 50% of estimated energy requirement for > 1 month
Abnormal prefeeding potassium, phosphorus, or magnesium serum concentrations[a]	Minimally low levels or normal current levels and recent low levels necessitating minimal or single-dose supplementation	Moderately/significantly low levels or minimally low or normal levels and recent low levels necessitating significant or multiple-dose supplementation
Loss of subcutaneous fat	Evidence of moderate loss	Evidence of severe loss
Loss of muscle mass	Evidence of mild or moderate loss	Evidence of severe loss
Higher-risk comorbidities (see Table S12)	Moderate disease	Severe disease

ASPEN, American Society for Parenteral and Enteral Nutrition; BMI, body mass index.
[a]Please note that electrolytes may be normal despite total-body deficiency, which is believed to increase risk of refeeding syndrome.

Minimally hypokalemia, hypophosphatemia, and hypomagnesemia were defined as the serum levels less than 3.5 mmol/L (3.5 mg/dL), 0.8 mmol/L (2.5 mg/dL), and 0.74 mmol/L (1.8 mg/dL), respectively, based on hospital reference for adults. Moderately/significantly hypokalemia, hypophosphatemia, and hypomagnesemia were defined as the serum levels less than 3.0 mmol/L (3.0 mg/dL), 0.6 mmol/L (1.8 mg/dL), and 0.5 mmol/L (1.2 mg/dL), respectively.
Patients were classified as being at high risk of RFS when scored ASPEN ≥ 1 score.
All grey overlays had not been utilized for scoring in these tables.

Table S7. Diseases and Clinical Conditions Associated With an Increased Risk of Refeeding Syndrome.

Chronic alcohol or drug use disorder
Dysphagia and esophageal dysmotility (eg, eosinophilic esophagitis, achalasia, gastric dysmotility)
Eating disorders (eg, anorexia nervosa)
Food insecurity and homelessness
Failure to thrive, including physical and sexual abuse and victims of neglect (particularly children)
Hyperemesis gravidarum or protracted vomiting
Major stressors or surgery without nutrition for prolonged periods of time
Malabsorptive states (eg, short-bowel syndrome, Crohn’s disease, cystic fibrosis, pyloric stenosis, maldigestion, pancreatic insufficiency)
Cancer
Advanced neurologic impairment or general inability to communicate needs
Postbariatric surgery
Postoperative patients with complications
Prolonged fasting (eg, individuals on hunger strikes, anorexia nervosa)
Refugees
Protein malnourishment

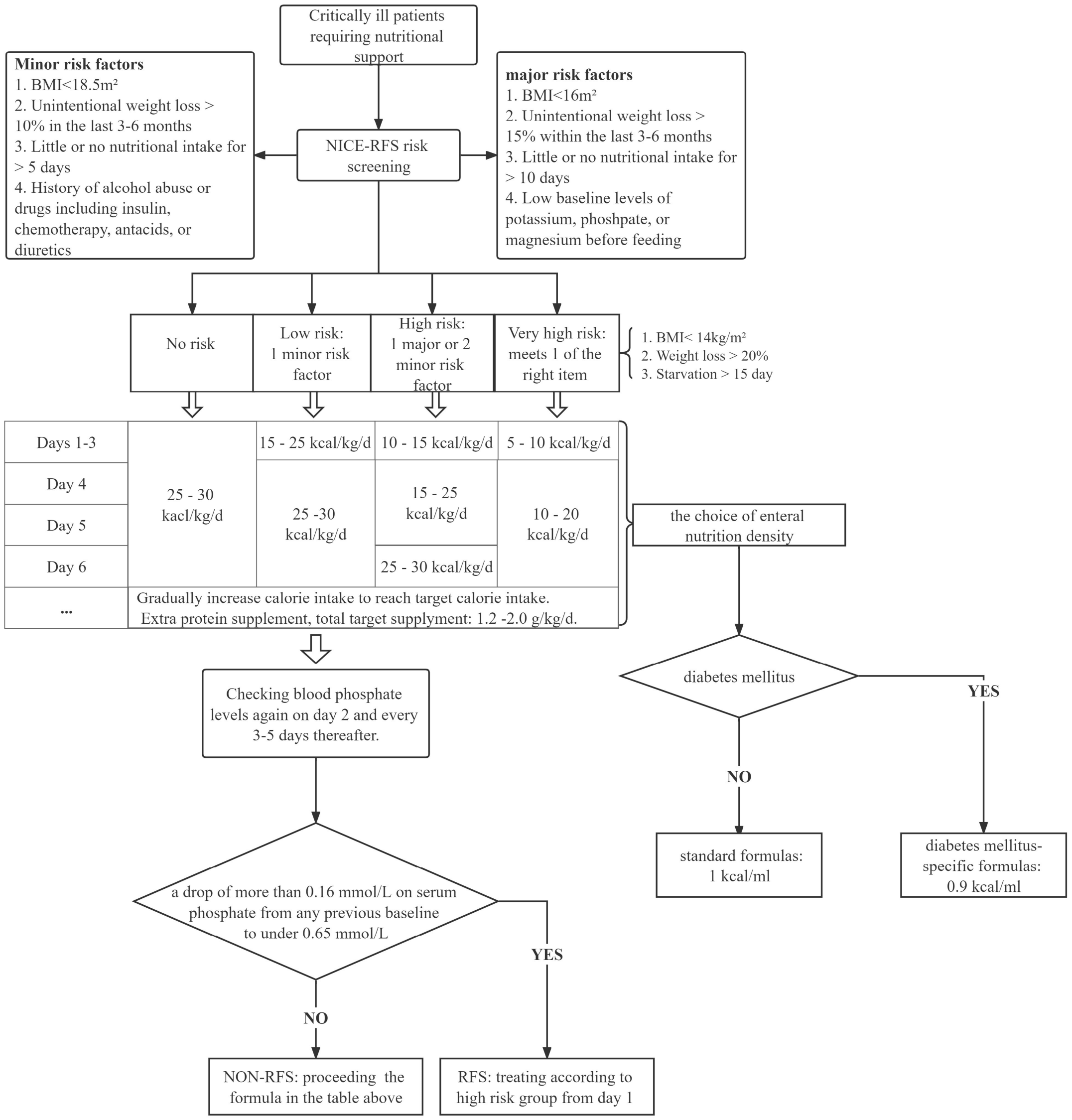

Figure S1. The refeeding protocol for neurocritically ill patients.

Table S8. Logistic regression analysis to identify predictors of RFS (Model B).

Parameter	Univariable analysis			Multivariable analysis		
	OR	95%CI	*P*	OR	95%CI	*P*
Hypertension	1.745	1.074-2.834	0.025	— —	— —	— —
Heart disease	1.921	1.023-3.606	0.042	— —	— —	— —
Day2 (Kcal/kg)	0.957	0.919-0.996	0.033			
APACHE II	1.069	1.028-1.111	0.001	1.046	1.003-1.090	0.034
Age	1.025	1.008-1.042	0.004	1.020	1.003-1.038	0.023
ASPEN	1.573	1.184-2.089	0.002	1.523	1.136-2.041	0.005

Day2 (Kcal/kg), day 2 caloric intake; APACHE II, Acute Physiology and Chronic Health Evaluation II; ASPEN, American Society for Parenteral and Enteral Nutrition; OR, odds ratio; CI, confidence interval.

Table S9. Logistic regression analysis to identify predictors of RFS (Model C).

Parameter	Univariable analysis			Multivariable analysis		
	OR	95%CI	*P*	OR	95%CI	*P*
Hypertension	1.745	1.074-2.834	0.025	— —	— —	— —
Heart disease	1.921	1.023-3.606	0.042	— —	— —	— —

Day2 (Kcal/kg)	0.957	0.919-0.996	0.033			
SOFA	1.120	1.032-1.215	0.006	1.097	1.009-1.192	0.030
Age	1.025	1.008-1.042	0.004	1.024	1.007-1.042	0.005
ASPEN	1.573	1.184-2.089	0.002	1.562	1.168-2.089	0.003

Day2 (Kcal/kg), day 2 caloric intake; ASPEN, American Society for Parenteral and Enteral Nutrition; SOFA, Sequential Organ Failure Assessment; OR, odds ratio; CI, confidence interval.

Table S10. Differences among the area under the receiver operating characteristic curves of SNAQ, GLIM, mNICE, ASPEN, and mASPEN.

	**SNAQ**	**GLIM**	**mNICE**	**ASPEN**	**mASPEN**
SNAQ		0.669	0.281	0.106	0.003
GLIM	0.669		0.388	0.105	<0.001
mNICE	0.281	0.388		0.284	0.003
ASPEN	0.106	0.105	0.284		0.005
mASPEN	0.003	<0.001	0.003	0.005	

AUC, area under the curve; SNAQ, the Short Nutritional Assessment Questionnaire; GLIM, the Global Leadership Initiative on Malnutrition developed a newer guideline; mNICE, an RFS risk group classification and the NICE RFS risk criteria; ASPEN, American Society for Parenteral and Enteral Nutrition; mASPEN, the combination of GCS, age and ASPEN to predict RFS; GCS, Glasgow Coma Scale.

Table S11. The efficiency of the ASPEN and different modifications of ASPEN in identifying RFS.

	**ASPEN**		**mASPEN1**		mASPEN2		mASPEN	
	%	**95%CI**	%	95%CI	%	95%CI	%	95%CI
Sensitivity	46.4	35.6-57.6	45.2	34.4-56.4	54.8	43.6-65.5	63.1	51.8-73.2
Specificity	76.4	71.8-80.4	77.7	73.2-81.6	67.5	62.6-72.0	64.0	58.9-68.7
PPV	29.5	22.1-38.2	30.2	22.5-39.1	26.4	20.2-33.8	27.2	21.2-34.1
NPV	87.0	82.9-90.3	86.9	82.9-90.2	87.5	83.1-90.9	89.0	76.8-92.3
Accuracy	63		72		65		64	
κ	12.5		19.1		15.7		17.8	

ASPEN, American Society for Parenteral and Enteral Nutrition; mASPEN1, combination of APACHE II and ASPEN; mASPEN2, combination of SOFA, age and ASPEN; mASPEN, combination of GCS, age and ASPEN; PPV, positive predictive value; NPV, negative predictive value; CI, confidence interval; κ: statistic, percent of agreement.

Table S12. Differences among the area under the receiver operating characteristic curves of ASPEN, mASPEN1, mASPEN2 and mASPEN.

	**ASPEN**	**mASPEN1**	**mASPEN2**	**mASPEN**
ASPEN		0.003	0.021	0.005
mASPEN1	0.003		0.994	0.510
mASPEN2	0.021	0.994		0.339
mASPEN	0.005	0.510	0.339	

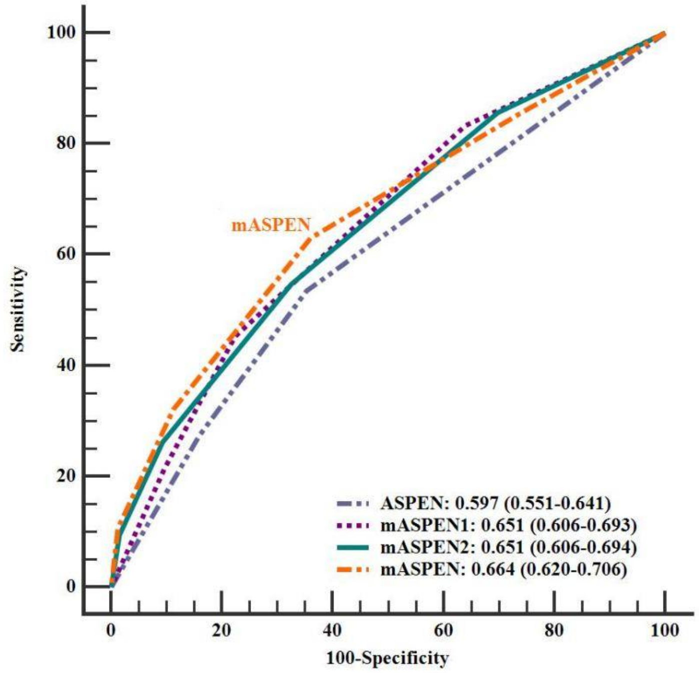

Figure S2. Receiver operating characteristic curve (ROC) analysis of ASPEN, mASPEN1, mASPEN2 and mASPEN scores for prediction of RFS. ASPEN, American Society for Parenteral and Enteral Nutrition; mASPEN1, combination of APACHE II and ASPEN; mASPEN2, combination of SOFA, age and ASPEN; mASPEN, combination of GCS, age and ASPEN.
